# Prior associations affect bumblebees’ generalization performance in a tool-selection task

**DOI:** 10.1016/j.isci.2022.105466

**Published:** 2022-10-31

**Authors:** Pizza Ka Yee Chow, Topi K. Lehtonen, Ville Näreaho, Olli J. Loukola

**Affiliations:** 1 Cognitive Ecology Research Group, Ecology and Genetics Research Unit, University of Oulu, Oulu, Finland; 2 School of Psychology, University of Chester, Chester, UK; 3 Natural Resources Institute Finland, Paavo Havaksen tie 3, 90570 Oulu, Finland

**Keywords:** Entomology, Cognitive neuroscience

## Abstract

A small brain and short life allegedly limit cognitive abilities. Our view of invertebrate cognition may also be biased by the choice of experimental stimuli. Here, the stimuli (color) pairs used in the match-to-sample tasks affected the performance of buff-tailed bumblebees (*Bombus terrestris*). We trained the bees to roll a tool, a ball, to a goal that matched its color. Bees trained with a yellow-and-orange/red stimuli pair took more training bouts to reach our color-matching criterion than those trained with a blue-and-yellow stimuli pair. When assessing the bees’ concept learning ability in a transfer test with a novel color, the bees trained with blue and yellow (novel color: orange/red) were highly successful, the bees trained with blue and orange/red (novel color: yellow) did not differ from random, and those trained with yellow and orange/red (novel color: blue) failed the test. These results highlight that stimulus salience can affect our conclusions on test subjects’ cognitive ability. Therefore, we encourage paying attention to stimulus salience (among other factors) when assessing the cognition of invertebrates.

## Highlights

• Results on cognitive abilities may be biased by the choice of experimental stimuli
• Bumblebees learned to choose and roll a ball that matched the color of a goal
• Performance in a generalization task depended on the colors we used in the training
• We encourage paying attention to experimental stimuli in studies of cognition

## Graphical abstract

**Figure:**
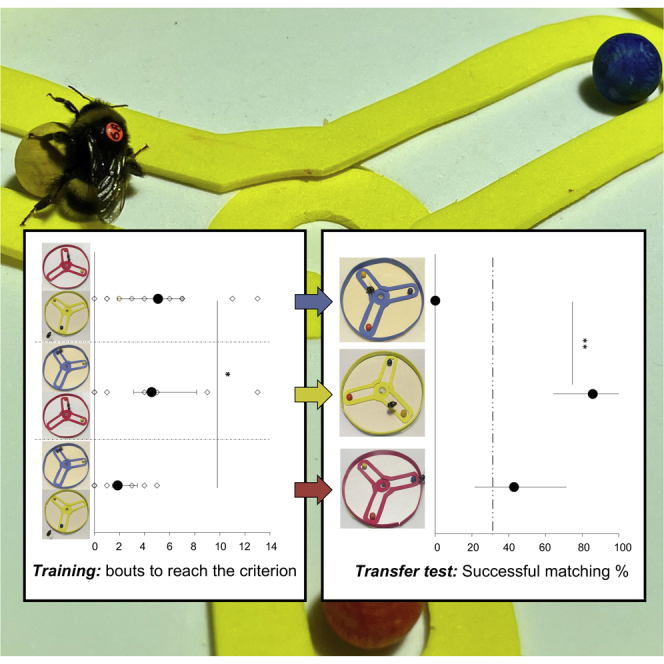

## Introduction

We have recently seen a rising interest in examining to what extent certain animal taxa possess aspects of “higher” cognition (e.g., tool selection, abstract concept, causal reasoning) that are comparable to those of humans. Because invertebrates have small brains and short lifespans,^1^^,^^2^ they were previously assumed to possess less sophisticated cognitive abilities. Contrary to this assumption, recent studies have shown that invertebrates, such as honeybees and bumblebees, exhibit remarkable performance in some cognitive tasks. For example, bees respond to external stimuli in flexible and sophisticated ways^1^^,^^2^ that include categorizing and counting objects^3^^,^^4^^,^^5^^,^^6^^,^^7^; discerning even arbitrary relationships between colors, shapes, and patterns^8^^,^^9^^,^^10^; and generalizing learned information in ways that may indicate the use of “concepts.”^11^^,^^12^^,^^13^ However, it remains unresolved to what extent these feats have been affected by extraneous factors, such as variation in the aspects of stimuli used in investigations (e.g., salience or properties that stand out or that would attract attention). Addressing these open questions will allow us to draw more solid conclusions and, thus, to minimize the risk of biasing our understanding of the cognitive abilities of invertebrates and the evolution of cognition.^14^

**Figure 1:**
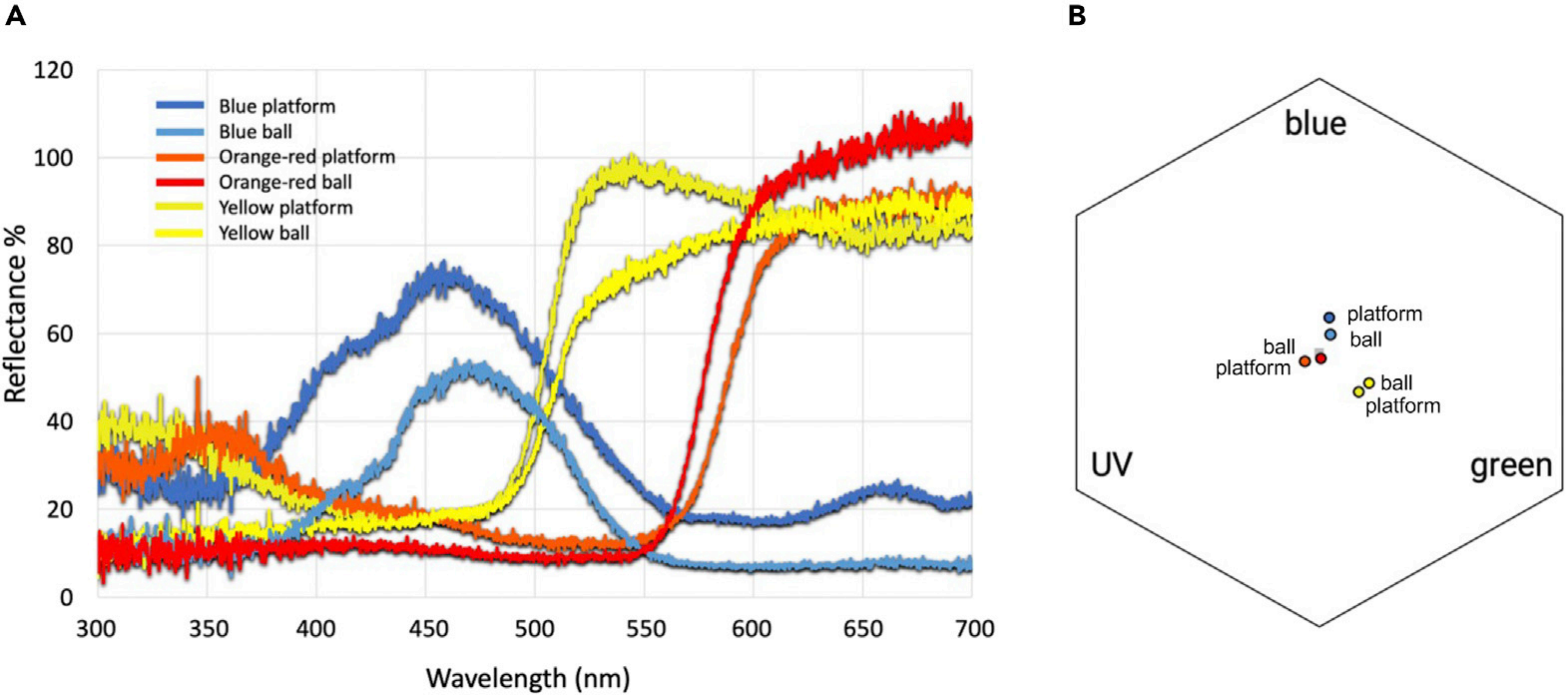
Spectral reflectance and color loci of the stimuli (A) Spectral reflectance and wavelengths of the platforms and balls used in the experiment. All reflectance was measured against a white background. (B) Distribution of color loci of platforms and balls in a hexagon color space to represent bee vision. Symbol colors correspond to those of (A).

Here, we used buff-tailed bumblebees (*Bombus terrestris*) to show that the conclusions drawn from an experiment may become drastically inconsistent when using stimuli of limited salience and variation. We demonstrated this by using three variations of a standardized test protocol when assessing bees’ concept learning ability, a higher cognition that can be measured by the well-established matching-to-sample paradigm (MTS).^15^ In each variation of the experiment (hereon, treatment group), we had a pair of stimuli with two different colors (i.e., blue and yellow, yellow and orange/red, and orange/red and blue). These color stimuli are within the perceptual range of bumblebees’ trichromatic color vision system,^16^ with bees’ photoreceptor spectral sensitivity peaking in the longer-wavelength regions (the UV, blue, and green color). The chosen colors had large distances from each other on the color reflectance spectrum (Figure 1). The blue color, as perceived by the human eye, has been shown to be the innate preferred color of most bumblebee populations,^17^^,^^18^^,^^19^ and the red color, as perceived by the human eye, appears as dark/shades to bees and, thus, is a more difficult color for them to perceive.^16^ This evidence suggests that bees may associate some colors with rewards faster than other colors. Indeed, some bumblebee species have been found to associate a reward with blue color faster than with other colors, such as yellow,^20^ even though preference for other colors can also be quickly induced by association^19^^,^^21^^,^^22^ and bumblebees can discriminate even between similar colors.^23^ Because the colors blue and yellow are frequently used as stimuli to examine bees’ behavior and cognition,^19^^,^^20^^,^^21^^,^^24^^,^^25^^,^^26^^,^^27^ it has remained unclear to what extent such color choices may affect the conclusions of the studies. Therefore, here, we included these two colors (that can be well perceived by bees), along with a much less frequently used color (orange/red which bees likely perceive as dark/shades^16^), to examine how the different stimulus pairs may affect bumblebees’ match-to-sample performance. We followed a typical MTS paradigm whereby individual bees first went through training, during which they learned an association (or a rule) by choosing one or more stimuli that matched with sample stimuli. The individuals’ concept learning ability was then assessed in a transfer test that required them to apply the learned association (or rule) when matching a novel stimulus. If the individuals successfully match the novel sample with the new stimulus, the performance can be attributed as “true abstract” concept learning.^15^ Note that the use of this MTS task taps into assessing concept learning within a single category (i.e., colors as a proxy of stimulus salience), which allows us to compare stimulus properties of this category when revealing the cognitive process of the bees in the transfer test.

When training the bees to match to sample, we followed an established ball-rolling experimental procedure.^25^ This procedure allowed the bees to demonstrate goal-oriented behavior (see below for details), meeting the basis of a higher cognitive ability—rule learning in the context of tool use. Accordingly, we assessed to what extent the bees exhibited higher cognitive processes beyond rule learning (i.e., true abstract concept learning^15^) when solving the MTS. Previous findings led us to hypothesize that stimulus salience (i.e., the chosen color pairs) would affect bees’ performance in the training^20^ and the subsequent transfer test.^21^ Specifically, the bees in different treatment groups would differ in their speed in reaching the training criterion, as well as in their ability to complete the transfer test. Such variation in the demonstration of concept learning would directly influence the conclusions drawn from the experiment.

**Figure 2:**
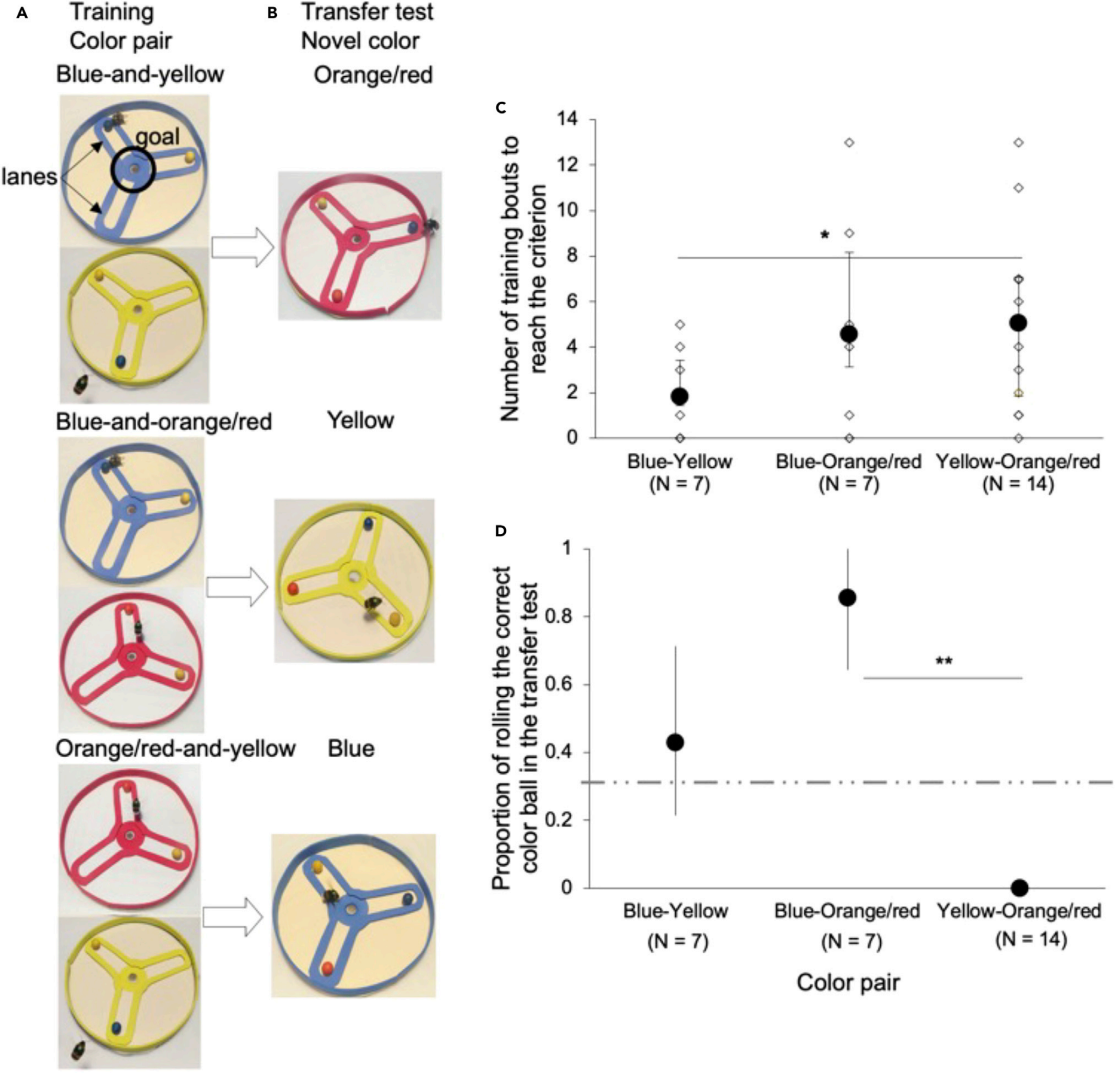
Experimental design and performance in the tasks (A) In the training, the three treatment groups, i.e., color pairs, were blue and yellow (top), blue and orange/red (middle), or yellow and orange/red (bottom). A bee was randomly assigned to a treatment group. The bee was presented with two balls of different colors, and she needed to roll the ball that matched the platform color along the lane to the goal in order to obtain the sucrose reward. (B) Once a bee completed the training, a ball with a novel color, as well as two balls with familiar colors, were presented to the bee in the transfer test; the bee was required to generalize the learned rule and roll the novel ball that matched with the platform color along the lane to complete the test. (C) The number of training bouts, shown here as bootstrap means (dark circles) and 95% confidence intervals and individual data (white diamonds), before reaching the criterion (i.e., these values exclude the 5 consecutive bouts of correct matching) for each color pair. ∗ p < 0.05 (generalized linear mixed model with a Poisson log link distribution). (D) The proportional success in rolling the correct ball (bootstrap mean and 95% confidence intervals) for each treatment group (color pair). The dashed light-grey line indicates success at random choice level. ∗∗p < 0.01 (Fisher exact test, Bonferroni corrected post hoc analyses).

We assessed the bees’ performance in both training and transfer test on a platform (see STAR Methods for details). This platform included three lanes, with the lanes being connected at a central point (“the goal”) and a bordering wall (hereafter, “platform”) (Figure 1). Given that we used three different pairs of colors, we had three treatment groups during the training. The platform was always one of the two “sample” colors used in that treatment (Figure 2A), and we randomly switched between the two platform colors every 1-3 bouts. During the training (5 min per bout), we placed two balls of different colors (e.g., blue and yellow: Video S1), each at the end of a different lane (Figure 2A). To successfully complete the training, it was not sufficient for a bee to simply associate the ball-rolling behavior with a reward (sucrose solution). The bee also had to learn a rule, “select the tool (the ball) that matches the platform’s color.” If the bee selected the correct tool (rolled the correct ball to the goal), we rewarded it immediately at the goal with 30% sucrose solution *ad libitum* (>200 μL) using a syringe. If the bee did not successfully perform the task, we used a model bumblebee to demonstrate how to obtain the reward. This is a shaping process that was developed by Loukola and colleagues^25^ (see STAR Methods for details, also see Video S2).

Document S1. Supporting information videos

Video S1. A bee rolling a ball in a training bout, Related to STAR Methods Video shows a bumblebee rolling a blue ball that matches the color of the platform and the goal. The bee lands on the platform, grasps the blue ball with her fore and middle legs and walks backwards with her hind legs. When the bee finally rolls the ball to the goal, she consumes the sucrose solution

Once the bee had successfully reached the training criterion of rolling the correct ball to the goal for ≥five consecutive bouts when choosing between the two available options (exact binomial probability for 5 out of 5 correct choices: p ≤ 0.031), we assessed whether the bee demonstrated concept learning in a transfer test.^15^ In this test, the bee was presented with three balls of different colors. One of the balls and the platform had a matching color that was novel to the bee, whereas the other two balls had the same colors as were used during the training (i.e., an orange/red-colored ball was used as the novel stimulus for the blue-and-yellow treatment group, a yellow ball for the blue-and-orange/red treatment group, and a blue ball for the yellow-and-orange/red treatment group) (Figure 2, Video S3). The balls were randomly placed at the end of a lane, and the bee was required to select the correct tool (ball). This test lasted 10 min, and we terminated the test if the bee had rolled the correct ball to the goal or 10 min passed, whichever came first. We considered the choice of the bee as “correct” if it rolled the ball of the novel color to the goal, without doing so with one of the other two balls first.

Video S2. Using a model bee in the training, Related to STAR Methods Video shows a bumblebee observing a model bee that is controlled by an experimenter. The model bee moves the ball that matches the color of the goal (orange-red) to the hole. As soon as the ball enters the hole, the experimenter (off screen) gives the sucrose solution as a reward for the bee. Note that the observer bee follows the model bee and the ball closely and drinks the sucrose solution directly after the demonstration

## Results

### Training

We used 28 bumblebees from 7 different colonies, with each treatment group having bees from two or more colonies. The number of training bouts that the bees took to reach the training criterion (5 successive successful bouts and the correct ball rolled) was 2 (range: 1-6, excluding the 5 successful bouts needed to reach the criterion), 6 (range: 1-15), and 6 (range: 0-13) in the blue-and-yellow (N = 7), blue-and-orange/red (N = 7), and yellow-and-orange/red (N = 7) treatment groups, respectively. Here, the yellow-and-orange/red treatment group took significantly more training bouts than the blue-and-yellow treatment group to reach the criterion (95% confidence interval [CI] 0.52 to 0.92; generalized linear mixed model [GLMM]: Z = 2.07, p = 0.038), whereas there were no significant differences between other treatment groups; (blue-and-yellow vs. blue-and-orange/red: 95% CI 0.41 to 0.88, Z = 1.36, p = 0.173; yellow-and-orange/red vs. blue-and-orange/red: 95% CI 0.19 to 0.65, Z = −0.78, p = 0.438, Figure 2C).

### Transfer test

Assuming a successful but random choice of color in the transfer test, the focal bee should have a probability of one-third to successfully match the ball and platform of the novel color. In the blue-and-yellow treatment group, 3 of the 7 bees (43%, bootstrap 95% CI 0.2 to 0.71) successfully rolled the ball that matched the novel color (orange/red) of the goal. This success rate was not significantly different from the expected (two-tailed binomial test, p = 0.879). In the blue-and-orange/red treatment group, 6 of the 7 bees (86%, bootstrap 95% CI 0.6 to 1) successfully matched the ball and goal of the novel color (yellow), the success rate being significantly better than expected (assuming successful tool use that is random with respect to color) (p = 0.010). Finally, none (0%, bootstrap 95% CI 0 to 0) of the bees in the yellow-and-orange/red treatment group (N = 7) succeeded in matching the ball and goal of the novel (blue) color in the test. Hence, these bees did significantly worse than expected (p = 0.019). We then replicated the procedure with another yellow-and-orange/red treatment group (N = 7) and got very similar results (median: 6, range: 1-11, test success: 0%; the two yellow-and-orange/red treatment groups combined: p = 0.003).

The three groups differed significantly in their success rate (proportion) in the test (Fisher’s exact test: p < 0.0001, Figure 2D). Post hoc analyses comparing the test performance between any two treatment groups (using the Bonferroni adjusted significance threshold of α = 0.017) showed that the yellow-and-orange/red treatment group did significantly worse than the blue-and-orange/red treatment group (Fisher’s exact test with Bonferroni correction: p = 0.0001), whereas no significant differences were detected between the other treatment groups (yellow-and-orange/red vs. blue-and-yellow treatment group: p = 0.026; blue-and-yellow vs. blue-and-orange/red treatment groups: p = 0.266).

**Figure 3:**
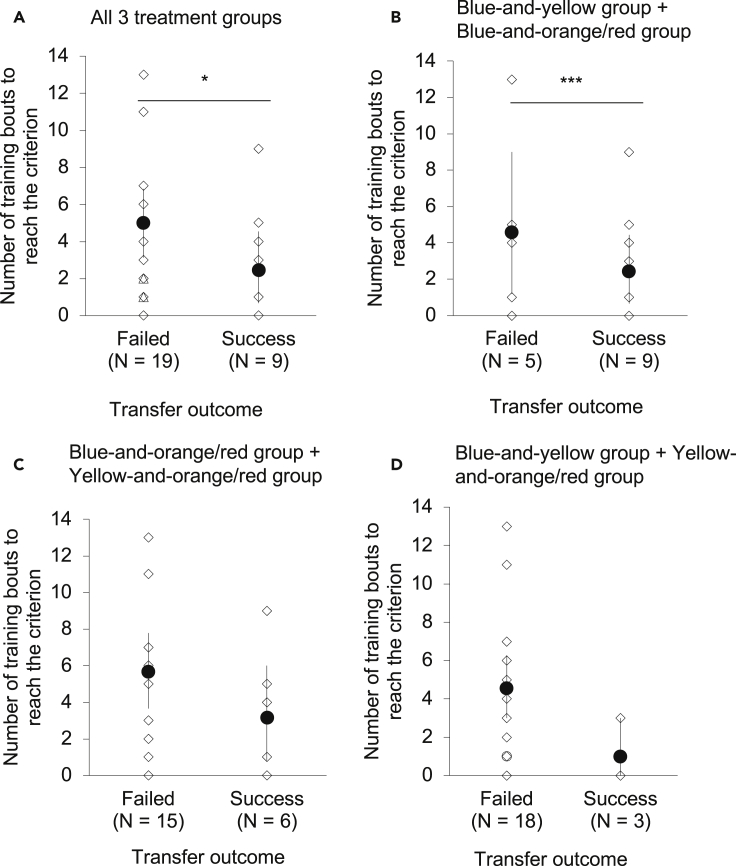
Comparison of the number of training bouts between the bees that successfully completed the transfer test with the bees that failed it The number of training bouts, shown here as bootstrap means (dark circles) and 95% confidence intervals and individual data (white diamonds), before reaching the criterion (these values exclude the 5 consecutive bouts of correct matching). (A) All three treatment groups (blue and yellow, blue and orange/red, and yellow and orange/red). (B) The blue-and-yellow treatment and blue-and-orange/red treatment groups only. (C) The blue-and-orange/red treatment and yellow-and-orange/red treatment groups only. (D) The blue-and-yellow treatment and yellow-and-orange/red treatment groups only. ∗p < 0.05, ∗∗∗p < 0.001 (generalized linear mixed model with a Poisson log link distribution).

To further examine the factors that may affect the bees’ test performance, we first compared whether the number of training bouts differed between the bees that successfully completed the test and the bees that failed the test. We found that the bees that successfully completed the transfer test took significantly fewer training bouts than the bees that failed the test (95% CI 0.12 to 0.48, GLMM: Z = −2.12, p = 0.034, Figure 3A). Given that the treatment groups differed significantly in the number of training bouts they took to reach the criterion, as well as in their test performance (Figures 2C and 2D), we further examined the effect of the number of training bouts that were needed to reach the training criterion on the test performance (with Bonferroni adjusted significance threshold of α = 0.017). To do this, we included the number of training bouts of two treatment groups in a model. We found that the effect of the number of training bouts on transfer success was significant when including the blue-and-yellow and blue-and-orange/red treatment groups (Figure 3B), with the bees that successfully completed the transfer test taking significantly fewer training bouts than the bees that failed the test (95% CI 0.12 to 0.38, Z = 3.23, p = 0.001), whereas other group comparisons were not significant (blue-and-orange/red treatment group vs. yellow-and-orange/red treatment group: 95% CI 0.14 to 0.55, Z = −1.54, p = 0.124; blue-and-yellow treatment group vs. yellow-and-orange/red treatment group: 95% CI 0.04 to 0.49, Z = −2.05, p = 0.045, Figures 3C and 3D).

**Figure 4:**
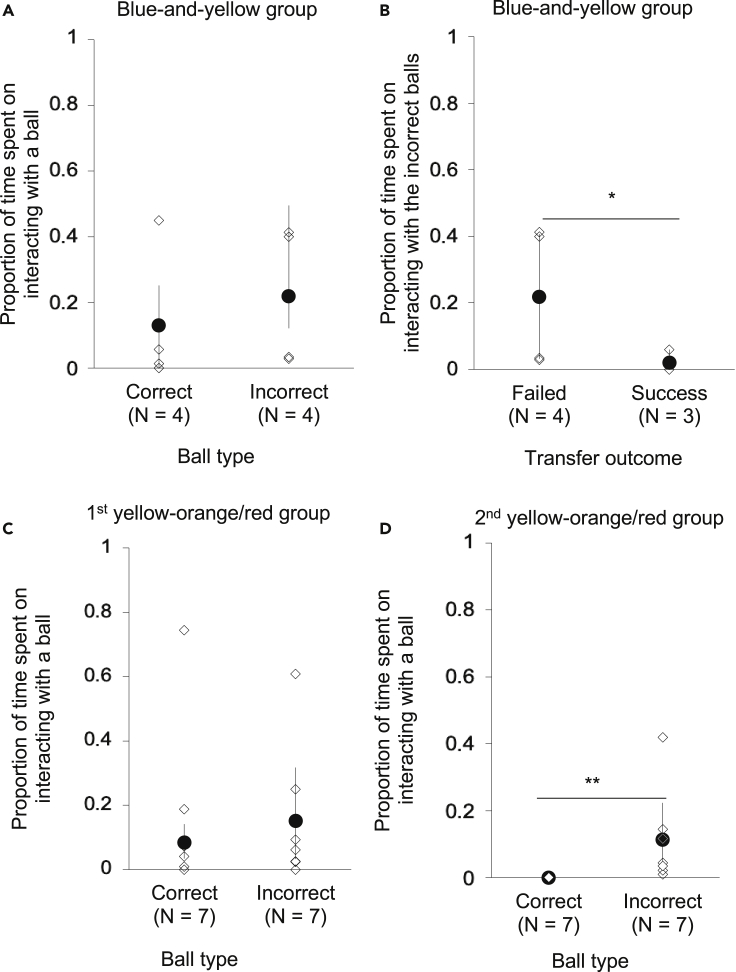
Analyses on individuals that failed the test (A–D) Bootstrap means (dark circles) and 95% confidence intervals and individual data (white diamonds). The proportion of time spent on interacting with the correct ball and incorrect balls (A) by the bees in the blue-and-yellow treatment group, (B) by the bees that failed the test and the bees that succeeded the test in the blue-and-yellow treatment group, and (C) by the first group and (D) by the second group of the yellow-orange/red treatment. ∗p < 0.05, ∗∗p < 0.01 (beta regression).

We also examined the behavior of the bees by measuring the time they were interacting with (i.e., touching or handling using their legs) the correct and incorrect balls in the transfer test. Specifically, we recorded the time from when a focal bee landed on a ball or otherwise touched it until it flew away from the ball. Focusing on the bees of the blue-and-yellow treatment group that failed the test (N = 4), there was no significant difference in the proportion of time spent interacting with the correct and incorrect balls (95% CI 0.07 to 0.61, beta regression: Z = −1.39, p = 0.163, Figure 4A). However, these bees did spend more time interacting with the incorrect balls than the bees that passed the test (N = 3) (95% CI 0.03 to 0.41, Z = −2.40, p = 0.016, Figure 4B).

In the first yellow-and-orange/red treatment group (N = 7), the bees did not significantly differ in the proportion of time touching the correct versus the incorrect balls (95% CI 0.23 to 0.72, beta regression: Z = −0.24, p = 0.812, Figure 4C), whereas the bees of the second group (N = 7) spent significantly more time touching the incorrect balls than the correct ball (95% CI 0.04 to 0.25, Z = −3.93, p < 0.0001, Figure 4D).

## Discussion

The findings support our hypothesis that the stimulus pair used for assessing bees’ concept learning ability affects their training and test performance, resulting in inconsistent conclusions. The stimulus pair with blue and yellow colors is frequently used when studying bees’ cognition, and our results on this treatment group suggest individual variation in concept learning, with the performance of the bees not being significantly different from random. In contrast, the high performance of the bees in the blue-and-orange/red treatment group provided strong evidence for concept learning, whereas the failure of both yellow-and-orange/red groups would indicate a lack of concept learning.

A common practice in examining cognition in invertebrates is to use one type of stimulus (e.g., color), and little attention has been given to stimulus salience and variation. Stimulus type and salience are important because they can draw individuals’ attention and guide their behavior, potentially affecting learning performance.^28^^,^^29^ Considering stimulus salience as colors, another bumblebee species has been shown to associate a reward with the color blue faster than with any other colors, such as yellow.^20^ Here, we further showed that the bees in the blue-and-yellow treatment group took fewer training bouts to reach the criterion than the bees in a treatment group without the blue color (i.e., the yellow-and-orange/red treatment group). Allegedly, the fast learning of the bees in the blue-and-yellow treatment group might be explained by a natural preference for blue, which could draw their attention and allow them to quickly associate blue with reward.^17^^,^^18^^,^^19^ However, this seems unlikely to be the case in our experiment because the bees in a treatment group that had a stimulus pair with blue (i.e., the blue-and-orange/red treatment group) performed similarly to those bees of the treatment group that had no color blue (the yellow-and-orange/red treatment group). With these results in mind, the treatment group differences in training performance (measured as the number of training bouts needed to reach the criterion) cannot be fully explained by other factors, such as the contrast between each color and the white background or the relative difference between the colors on the reflectance spectrum. Regarding the latter possibility, the color stimuli that had a short distance from each other on the reflectance spectrum (yellow and orange/red) might have impeded the bees’ concept learning, and thus impaired their performance during the training. However, we consider it unlikely that the reflectance spectrum distance played an important role because the number of training bouts taken to reach the criterion was comparable between the bees in the treatment group that had the farthest distance between the two colors (blue and orange/red) and the shortest distance (yellow and orange/red) on the reflectance spectrum.

The color of the stimulus pairs, as a proxy of stimulus salience, had a pronounced effect on the test performance. Hence, we argue that the differences in the transfer test performance relate to an aspect of the stimulus: the relative reflectance distance between the colors in the training and the novel color in the test. In the test, a short distance between the novel color (yellow) and one of the colors that the bees got familiar with during the training (orange/red) probably made it easier for the bees in the blue-and-orange/red treatment group to approach the novel color, resulting in a highly successful performance. The high success of this treatment group may also be partially explained by the increased length of training, which has been shown to be the case in honeybees.^30^ However, we do not think this is the case because our results reveal the opposite trend. The results also suggest that the effect of training duration on the transfer outcome may depend on the specific stimulus color pairs that the bees experienced during the training; the bees in the yellow-and-orange/red treatment group and the blue-and-orange/red treatment group had comparable training experience, but both yellow-and-orange/red and treatment groups failed the test completely, whereas the bees in the blue-and-orange/red treatment group had the highest success rate in the test. Overall, the different aspects of stimulus salience, together with the details of the experimental procedure, may have had accumulated effects on the bees’ match-to-sample training performance. Nevertheless, these possibilities have rarely been investigated or reported in studies of cognition.^31^ Moreover, the high success of the blue-and-orange/red group is unlikely to demonstrate true abstract concept learning, but rather is likely to indicate that bees approach stimuli that are similar to familiar ones,^21^^,^^32^ for instance, due to generalization of stimulus-response chains, which has been previously demonstrated in pigeons.^33^

The behavior of the bees that failed the test further highlights the effect of stimulus salience on test performance. Failing to transfer a learned rule to a novel stimulus could be due to a number of reasons. In our case, the test performance of the bees in the blue-and-yellow treatment group not being significantly different from random suggests that some individuals did not simply follow the generalization stimulus-response chains. The fact that these individuals spent a comparable proportion of time interacting with the correct and incorrect balls suggests that they may have been confused in the novel context. Similarly, confusion might contribute to the test performance of the bees in the yellow-and-orange/red treatment group. The poor performance of both yellow-and-orange/red groups may also indicate perseverance on learned information, as the bees interacted with the incorrect balls significantly longer than the correct balls. Despite this, the difference we detected in handling the correct versus incorrect balls between the first and second yellow-and-orange/red groups may be related to the between-individual variation in bumblebees.^34^^,^^35^ The two yellow-and-orange/red groups had a similar trend in interacting with the balls, and none of the bees passed the transfer test. While the failure of transfer in this treatment group could suggest the absence of concept learning, or that the bees failed to switch from a cold color (blue) to a warm color (yellow or orange/red), the failure of the bees in both yellow-and-orange/red groups is more likely explained by neophobia^36^: None of them approached the novel blue ball in the test. Such a response may be explained by the farther spectral distance (i.e., dissimilarity) between the familiar colors and the novel color in that treatment group, resulting in the bees failing to apply the learned rule in a novel context.

To conclude, bees trained with different stimulus pairs greatly differed in their success rate of completing the transfer test, highlighting the need for using a range of stimulus pairs when assessing the cognitive abilities of invertebrates. The common choice of using color as a stimulus over other parameters, such as shape and size, relates to the significant role that (floral) colors play in bees’ life history as pollinators.^37^^,^^38^ However, floral features (such as shape or size) also vary in nature, and invertebrates have been shown to use multiple cues (colors, patterns, shapes, and odors) when foraging.^39^ Therefore, future studies should use a range of stimuli within a single category alongside various stimulus types over different experiments when assessing invertebrate cognition. To our knowledge, there is only one study to date demonstrating that insects, particularly honeybees, show concept learning.^4^ Accordingly, we urge future studies to consider varying stimulus types to increase the relevance of investigations of invertebrate cognition.

### Limitations of the study

It is possible that there were differences between treatment groups that remained undetected, due to the relatively small sample size. In particular, small sample sizes imply that effect sizes need to be large to be detected (and it is possible for larger effect sizes to appear significant by chance alone than when large sample sizes are used). While the effects of stimulus pair on the bees’ matching-to-sample training and test performance valid evidence, we underscore the need to consider the potential effects of extraneous factors, such as stimulus presentation duration and intertrial interval, on the conclusions when examining the cognitive ability of interest.

### Data and code availability

Our data are available on Dryad: https://doi.org/10.5061/dryad.tqjq2bw36.

## STAR★Methods

### Key resources table

**Table:** 

RESOURCE	SOURCE	IDENTIFIER
**Deposited data**		

Raw and analyzed data	Dryad	https://doi.org/10.5061/dryad.tqjq2bw36

**Experimental models: Organisms/strains**		

Buff-tailed bumblebee (*Bombus terrestris*)	Koppert B.V., The Netherlands	https://www.koppert.com/natupol-smart/

**Software and algorithms**		

R (version 3.6.2)	R Core Team	https://www.Rproject.org/

**Other**		

Pollen (food supplementary)	Koppert B.V., The Netherlands	https://www.koppert.com/additives/
Video recorder	Sony Xperia XZ Premium	https://www.sony.com/electronics/support/mobile-phones-tablets-mobile-phones/xperia-xz-premium/specifications
Number tags	Bienen-Voigt & Warnholz, Germany	https://www.bienen-voigt.de/en/beekeeper-products/marking-discs-1-99-white opalith-weiss2011
Color markers	Uni POSCA PC-5M, Mitsubishi Pencil Co., LTD. Japan	https://www.posca.com/en/product/pc-5m/

### Resource availability

#### Lead contact

Further information and requests for resources should be directed to, and will be fulfilled by, the lead contact, Olli J. Loukola (olli.loukola@oulu.fi).

#### Materials availability

Besides data and R codes (below), this study did not generate any new reagents or materials.

### Experimental model and subject details

In this study, we investigated the effect of stimulus salience (color as a proxy) on the matching-to-sample performance of an insect species, the buff-tailed bumblebee (*B. terrestris*). The experiments were carried out in accordance with the ethical guidelines of the Association for the Study of Animal Behavior (UK) and the Animal Behavior Society (USA). We also strictly followed all laws and regulations of Finland where studies on invertebrates do not require a specific license/permit.

### Method details

#### Study system

The experiments were conducted in 2018 in bumblebee facilities at the Botanical Garden of the University of Oulu, Finland. We obtained bumblebees from a continuous rearing program (Koppert B.V., The Netherlands). Each of the bumblebee hives (N = 7) used in the study was housed in a wooden box (31 × 13.5 × 11.5 cm) that had holes for air exchange and separated entrance and main hive chambers, with a 3 cm layer of cat litter at the bottom of the former. Each hive had a queen and ∼30 workers. We provided each hive with ∼7 g commercial pollen (Koppert B.V., The Netherlands) on every second day and, when not being trained or tested (see below), the bees had a continuous opportunity to forage on a 30% sucrose solution from a feeder.

We used one hive at a time. Its entrance chamber was connected to a transparent plexiglass corridor (25 × 4 × 4.5 cm), which allowed the bees to access an arena (60 × 25 × 43 cm). Three transparent plastic sliding doors along the corridor provided means to control the access of bumblebees to the arena (for testing purposes). This setup was used during pretraining, training and testing (see below).

#### Pretraining

The aim of this pretraining was to allow the bees to learn the location where to access the reward. In the pretraining, the bumblebees had unrestricted access to the arena where they could access 30% sucrose solution from the middle of a circular white platform (Ø 150 mm) that was placed in the central part of the arena. During the pretraining, the most active foragers were identified by an observer (OJL and VN) and each of these bees was marked with a small number tag. These tagged individuals were used in the training and test.

#### Training

The purpose of the training was to assess our hypothesis, while training the bees to match to a sample. In the training, the center of the arena had a white circular plastic platform (Ø 150 mm with a bordering wall 12 mm high). This platform had a hole in its center (Ø 12 mm) and a colored circular zone encompassing the center hole (Ø 35 mm). Three lanes (20 mm wide at the center section, outlined by 1 mm high and 10 mm wide plastic strips) ran from the rim of the platform and converged at the central zone at 120° angles relative to the adjacent lanes (Figure 1A). The platform also had two wooden balls (Ø 8.5 mm) of different colors (blue and yellow, blue and orange/red, or yellow and orange/red), painted using Uni POSCA PC-5M, Mitsubishi Pencil Co., LTD. Japan, Figure 1). The bordering wall, the three lanes, the circular zone around the center hole (collectively referred to as ‘platform’), and one of the two balls matched the platform color, while the other ball was of a different color.

During the training, only one tagged bee (N = 28 over the experiment) was allowed to access the arena at a time. Each bee was randomly assigned to a treatment group (blue and yellow, blue and orange/red, or yellow and orange/red) and thus only exposed to only two of the three colors used in the experiment. In each treatment group, a bee was exposed to the platform of two different colors. There were two balls, with one of the two balls always matching the color of the platform and the other ball having the other color. Each bee was challenged with a color matching task in the context of token use (see^25^). The bee was given 5 min to complete a training bout. During a training bout, the 'correct' (rewarding) action required the bumblebee to successfully roll the ball that matched the color of the platform, from the rim of the platform to its center hole (Video S1). Rolling the ball onto the central zone surrounding the hole, but not all the way into the hole, was also considered as successful. If the bee was successful, the experimenter used a syringe to immediately place a reward of 30% sucrose solution *ad libitum* (>200 μL) in the central hole for the bee to drink. Failing to accomplish the task within 5 min (or rolling the non-matching ball onto the central zone) was deemed as ‘incorrect’ (i.e., the bumblebee did not accomplish the task). After each training bout (successful or fail), the bumblebee was allowed to use the connecting corridor to visit the hive and then later to return to the arena to try again (i.e., the start of another training bout). We cleaned both balls and the platform with ethanol to neutralize any odor cues after each training bout. We also switched the color of the platform between the two color options for that bee after every 1-3 training bouts. The behavior of the bee was video recorded for behavioral analysis using a Sony Xperia XZ Premium smartphone. The bee participated in the transfer test as soon as she had reached the criterion of training (matching the ball that had the color as the platform for 5 or more consecutive bouts). At the end of each day, all the bees were allowed to freely access the arena to forage from a white platform, as during the pretraining phase.

The training progressed in a stepwise fashion that included four steps. In the first step, the ball that matched the color of the platform was already in the central hole, and the bumblebee was rewarded as soon as it touched that ball. Once this had happened, the task progressed to the second step, in which the 'correct' ball was placed next to the central zone, from where the bee had to roll the ball into the hole. After the bee had successfully completed this step, the third step involved the bee rolling the ball that was being placed midway between the central zone and the rim of the platform to the goal. When the bee completed this step, the final step involved both balls being placed at the rim of the platform, from where the bee needed to roll the ‘correct’ ball to the center. Most of the bees failed one or more steps during the training. When a bumblebee did not successfully perform the task correctly within a 5-min training bout, the experimenter (OJL and VN) used a plastic model bumblebee (which mimicked the color patterns of a *B*. *terrestris* worker) that was attached to a thin transparent stick to demonstrate how to solve the task.^25^ The experimenter then used a syringe to give the sucrose solution directly to the bee (Video S2). A model, rather than living, bumblebee demonstrator ensured a desired and standardized demonstration.

#### Transfer test

The purpose of the transfer test was to test our hypothesis while assessing whether the bees exhibit concept learning by applying a learned rule in a novel context. The test was conducted once a bee reached the training criterion (5 or more successful training bouts in a row). The test consisted of a single bout that was similar to the last step of training with the following exceptions: The platform was of the 'third' color that the bumblebee had not encountered during the training. In addition, the platform had 3 balls of different colors: blue, yellow or orange/red. One ball was placed at the end of each lane, next to the rim of the platform (Figure 1B, Video S3). The test ended if the bee rolled the correct ball to the central hole. If the bumblebee rolled a ball of a color that did not match with that of the platform, it was considered to have failed the test and the test continued during which the ‘incorrect’ ball was returned to the trim of the platform until 10 min passed.

### Quantification and statistical analysis

All statistical analyses were conducted using R version 3.6.2^40^ and SPSS v25 (IBM Corp). Generalised Linear Mixed Models (GLMM) with a poisson distribution (link = log) in the package 'glmmTMB'^41^ were used to examine whether the color pair (three levels: blue-and-yellow, blue-and-orange/red, and yellow-and-orange/red) affected the number of bouts taken to reach the training criterion. We included bee ID nested within colony ID as the random variable.

To assess whether bumblebees learned to solve the generalization task, we used ⅓ as the baseline expectation and compared it to bumblebees’ performance (in terms of the number of bumblebees that solved vs. did not solve the task) for each of the treatment groups using a binomial test. To examine the effect of the color pairs in relation to success in the test, we conducted a GLMM with a binomial distribution (link = logit). However, due to convergent issues (likely related to the zeros, or the bees in the yellow-and-orange/red treatment group completely failing the test), the model could not be run. Accordingly, we compared bumblebees’ test performance between the three treatment groups using Fisher exact test. We also used Fisher exact test with Bonferroni corrections for posthoc analyses, when comparing the performances between any two treatment groups (adjusted significance level: p ≤ 0.017).

Another GLMM with Poisson distribution (link = log) was conducted to examine whether the number of training bouts differed between the bees that successfully completed the transfer test and those bees that failed the test. For this analysis, we included the colony, bee ID and color pairs as the random variables. Given that the treatment groups differed in the number of training bouts taken to reach the criterion (Figures 2C and 2D), we further conducted three GLMMs with Poisson distribution (link = log) to examine whether the bees that had successfully completed the transfer test differed from those bees that had failed the test between treatment groups using separate analyses. In these models, we included colony and bee ID as the random variables. We adjusted the p values using Bonferroni correction (p = 0.017) for multiple comparisons.

To further examine why the bees in the blue-and-yellow group failed the test, we conducted beta regression using the package ‘betareg’.^42^ One analysis was conducted to examine whether the bees that failed the test spent a higher proportion of time interacting with the incorrect balls than the correct ball. Another analysis was conducted to compare whether the bees that failed the test spent a higher proportion of time interacting with the incorrect balls than the bees who succeeded the test. Beta regressions were also used to analyze the proportion of time spent on interacting with the balls for the two yellow-and-orange/red groups. We compared the proportion of time interacting with the incorrect balls and the correct ball for the first yellow-and-orange group in one analysis, and another analysis for the second yellow-and-orange group.

Other than the posthoc tests using Bonferroni corrections with adjusted p values, we consider each test reached a statistical significance when α < 0.05 (two tailed). All results reported here used bootstrapped methods (10000 random replicates) on raw data to obtain means and 95% confidence intervals.

To visualize the colors of the platforms and balls as perceived by bees (Figure 1B), we used the color hexagon model^43^ with the vismodel function using the package ‘pavo’^44^ in R.

## Data Availability

• Numerical raw data of this study and the original codes required to reanalyze the data are deposited at Dryad Digital Repository: https://doi.org/10.5061/dryad.tqjq2bw36. • Any additional information required to reanalyze the data reported here is available from the lead contact upon request.

## Supplemental information

Video S3. A bee rolling the ball in the transfer test, Related to STAR Methods Video shows a bumblebee choosing between balls of different colors in the transfer test. The bee lands on the yellow platform, checks the orange-red and blue balls, and finally rolls the yellow ball toward the yellow goal
