# Supplementary material for: Prior associations affect bumblebees’ generalization performance in a tool-selection task
Source: iScience. 2022 Oct 31;25(11):105466. doi: 10.1016/j.isci.2022.105466 (PMC9663899; doi:10.1016/j.isci.2022.105466)
Supplement: Document S1. Supporting information videos [file mmc1.pdf]

iScience, Volume *25*

Supplemental information

Prior associations affect bumblebees' generalization performance in a tool-selection task

Pizza Ka Yee Chow, Topi K. Lehtonen, Ville Näreaho, and Olli J. Loukola

Supplemental information

Supporting Information Videos

Descriptions of Supplemental Videos 1-3 can be found in the Method Details section of the main text.
